# Comparative efficacy of plant derived extracts with the insecticide mospilan on two whitefly species *Bemisia tabaci* biotype B and *Trialeurodes ricini*

**DOI:** 10.1038/s41598-024-84958-0

**Published:** 2025-01-15

**Authors:** Hanaa S. Hussein, Mamdouh H. Idriss, Farouk H. El-Gayar, Hebatullah Yehia Saad Mousa, Mohamed Z. M. Salem

**Affiliations:** 1 Department of Applied Entomology and Zoology, Faculty of Agriculture, Alexandria University, Alexandria, 21545 Egypt; 2 Forestry and Wood Technology Department, Faculty of Agriculture, Alexandria University, Alexandria, 21545 Egypt

**Keywords:** Acetylcholinesterase, GC-MS, Mospilan, Natural extracts, Repellency index, Whiteflies, Biological techniques, Developmental biology, Zoology

## Abstract

The insecticidal, synergistic, and acetylcholinesterase (AChE) inhibitory effects of plant *n*-hexane extracts (HEs) were evaluated. The HEs from thyme (*Thymus vulgaris* L.) leaves, garlic (*Allium sativum* L.) bulbs, and weeping willow (*Salix babylonica* L.) leaves were used in comparison with the acetamiprid insecticide (mospilan) against two whitefly species, *Bemisia tabaci* (Gennadius) (Hemiptera: Aleyrodidae) biotype B and *Trialeurodes ricini* (Genn.) (Hemiptera: Aleyrodidae). Furthermore, using the choice test design, the repellent efficacy of three extracts was investigated against whitefly *B. tabaci* biotype B. The chemical compositions of HEs were identified using gas chromatography-mass spectrometry (GC-MS) and gas chromatography with flame-ionization detection (GC-FID) analysis. The main compounds of thyme HE were thymol and geranyl-α-terpinene; in garlic bulbs HE were diallyl sulfide and allyl tetrasulfide; and in weeping willow HE were 6-phenyltridecane, 6-phenyldodecane, and 5-phenyldodecane, while the methylated fatty acids were stearic and palmitic. The HEs of weeping willow and garlic showed the maximum toxicity against *B. tabaci*, while the HEs of thyme and garlic showed the highest toxicity against *T. ricini*. Mospilan with HEs resulted in a potentiating effect, with co-toxicity factors ranging between 21.47 for a mixture of garlic HE + mospilan against *B. tabaci* and 37.65 for weeping willow HE + mospilan against *T. ricini*. The mix of mospilan + weeping willow HE recorded the highest acetylcholinesterase (AChE) inhibitory effect 48 h after treatment. The highest expulsion effect was recorded by 2% thyme HE, with a repellency index (RI) of 88.22%. The HE of weeping willow at 1% exhibited the highest attractant effect with an RI value of -8.94%. The current research lays the groundwork for the integrated pest management (IPM) of *B. tabaci* biotype B and *T. ricini* by employing natural extracts and pesticides blends.

## Introduction

Whiteflies cause significant yield loss, either directly by sucking plant sap or indirectly by excreting honeydew that encourages the growth of sooty fungi and vectoring plant viruses^1^. The sweet potato whitefly, *Bemisia tabaci* (Gennadius) (Hemiptera: Aleyrodidae), has been considered one of the most important insect pests, attacking a wide variety of agricultural commodities^1–5^, whereas, *B. tabaci* has been recorded to transmit several distinct groups of plant viruses, including geminiviruses, carlaviruses, potyviruses, closteroviruses, nepoviruses, luteoviruses, and DNA-containing rod-shaped viruses^6–9^. The silver leaf whitefly, *B. tabaci*, was reported to phosphorylate cyanogenic glucosides in cassava (*Manihot esculenta* Crantz)^10^.

The castor-oil whitefly, *Trialeurodes ricini*, is a polyphagous insect and commonly a pest of castor-oil plants (*Ricinus communis* L.)^11,12^, and many plants belonging to the families Caesalpiniaceae, Papilionaceae, and Mimosaceae. *T. ricini* was known as a vector of the tomato yellow leaf curl virus (TYLCV) in Egypt^13^, and Eastern India^14^. The heavy infestations with *T. ricini* reduced the growth of castor plants^15^.

The excessive use of chemical insecticides for whitefly management leads to the development of cross-resistance to many insecticides^16^. Therefore, there is an urgent need to develop alternative control methods^17–22^. Natural extracts have been evaluated previously for insecticidal activity and repellency effects against whiteflies and are recommended as alternatives for controlling this pest through suitable integrated pest management programs^23,24^. The expulsion effect of extracts from *Plectranthus neochilus* Schltr., *Ageratum conyzoides* L., and *Tagetes erecta* L. on *B. tabaci* makes them play a vital role in its control, consequently reducing transmission of plant viruses^24^. In addition, they have minimal effects on natural enemies^26,27^.

Several botanical extracts were observed insecticidal effects against *B. tabaci* eggs, nymphs and adults, i.e., *Petiveria alliacea* L. and *Trichilia arborea* C.DC. leaf ethanol^28^, *Azadirachta indica* A.Juss. seed oil^29–31^, *Lepidium sativum* L., *Achillea biebersteinii* Afan., or *Retama raetam* (Forssk.) Webb & Berthel^32^. , aqueous extracts from *Tradescantia pallida* (Rose) D.R.Hunt branches and leaves^33^, extracts from *L. sativum*, *Pimpinella anisum* L., *Galium longifolium* (Sibth. & Sm.) Griseb., *Retama raetam* (Forssk.) Webb & Berthel. and *Ballota undulata* (Benth.) Salmaki & Siadati^32^, neem azal-S extract^34^, and extracts of *Laurus nobilis* L., *Verbascum thapsus* L., *Tanacetum vulgare* L., and *Artemisia vulgare* L^35^.

Tomato plants dipped in 10% (wt/wt) of extracts from *Ruta chalepensi*s L., *Peganum harmala* L., and *Alkanna strigosa* Boiss. & Hohen. and infested with whiteflies were effective in reducing the numbers of *B. tabaci* immatures, similar to imidacloprid treatment^35^. Different physiological modes of action of plant extracts and their components have been reported, i.e., inhibition of AChE and adenosine triphosphatases (ATPases)^37,38^.

This study aimed to test the insecticidal efficacy of three *n*-hexane extracts from garlic, thyme, and weeping willow against two whitefly species, *B. tabaci* biotype B and *T. ricini*, and evaluate the synergistic effect as well as the AChE inhibitory effect of these extracts when mixed with conventional insecticide, mospilan (acetamiprid). Additionally, the study aimed to investigate the repellent efficacy of the three extracts against whitefly *B. tabaci* biotype B.

## Materials and methods

### Sweet potato whitefly, *Bemisia tabaci* culture

The identification of the mother culture of the whitefly *Bemisia tabaci* Genn. biotype B was achieved by two world authorities of whiteflies: Miss Louise M. Russell of the U.S. National Museum, USDA, USA, and Dr. Lawrance A. Mound of the British Museum (Natural History), London, U.K. (cf. El-Helaly, 1966). The colony used in the present study was reared in the same place under the same conditions on tomato plants (*Solanum lycopersicum* L., Solanaceae), at 25 ± 7 °C, 65 ± 5% RH, and under natural light conditions^39^. The experiments were carried out during the summer season of 2018.

### Field strain of the castor-oil whitefly, ***Trialeurodes ricini***

**Fig. 1:**
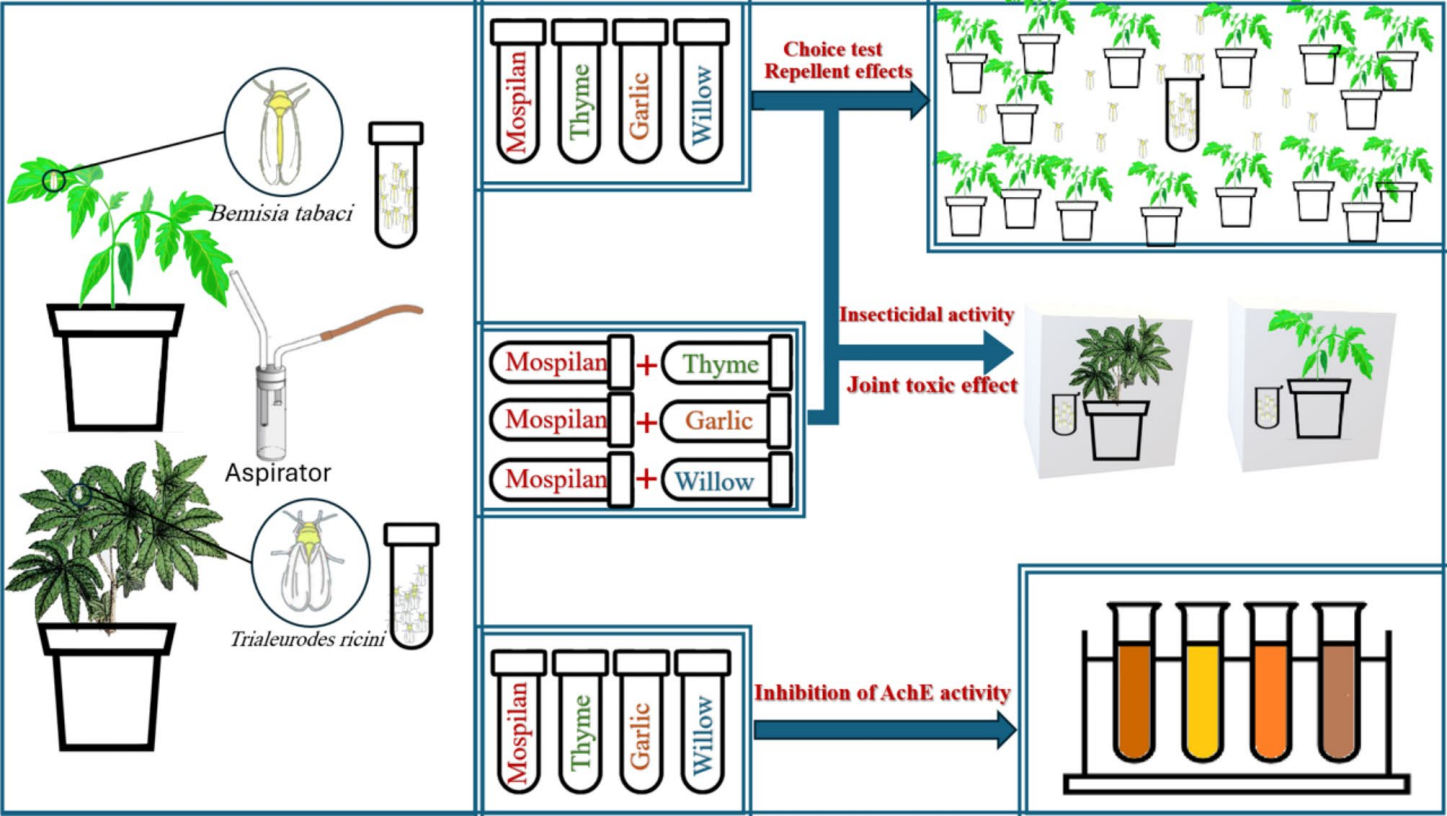
The insecticidal activity of the tested materials.

*Trialeurodes ricini* (castor-oil whiteflies) adults were collected using an aspirator (Fig. 1) very early in the morning from the infested castor fields in Hagar El Nwateia, Alexandria, Egypt, and the puparia were identified as *T. ricini* at the Faculty of Agriculture, Alexandria University, Egypt, according to the key of Martin et al.^40^. It should be mentioned that the castor whitefly *T. ricini* was collected from a clean, isolated region where no pesticide treatments are carried out, which means they are considered a sensitive strain.

### Plant extracts and the insecticide

Air-dried plant materials from thyme (*Thymus vulgaris* L.) leaves and garlic (*Allium sativum* L.) bulbs were obtained from some herbarium at Alexandria, Egypt, while weeping willow (*Salix babylonica* L.) leaves were obtained from the Faculty of Agriculture farm at Abies Station Farm, Alexandria, Egypt (31°12 N, 29°55E) under the permission of the Department of Applied Entomology and Zoology, Faculty of Agriculture, Alexandria University, Egypt. Acetamiprid 20% SP, a systemic insecticide (neonicotinoids) (Mospilan^®^) was used as a positive control with a field rate of 25 mg/L in water.

### Extraction of ***n***-hexane extracts and the GC-MS analysis

Air-dried plant materials, including thyme leaves, weeping willow leaves, and garlic bulbs, were ground to powder using a small laboratory mill. About 100 g of each material was separately extracted using *n*-hexane (150 mL) by soaking method^41^ for three days, then filtered using filter paper (Whatman No. 1) and concentrated by evaporating the solvent at 35 °C using a rotary evaporator and poured in Petri dishes to complete the dryness of extracts from the solvent. The *n*-hexane extracts (HEs) were analyzed for their chemical compounds using the gas chromatography-mass spectrometry (GC-MS) apparatus of the Focus GC-DSQ Mass Spectrometer (Thermo Scientific, Austin, TX, USA) with a TG-5MS direct capillary column (30 m × 0.25 mm × 0.25 μm film thickness). After being kept at 50 °C for two minutes, the temperature of the column oven was raised by 5 °C/min to 250 °C, and then it was raised to 280 °C (10 °C/min). We kept the temperatures of the injector and detector (MS transfer line) at 250 °C. As a carrier gas, helium was employed at a steady flow rate of 1 mL/min. An Autosampler AS3000 connected to a split mode GC automatically injected diluted samples of 1 µL after a 4 min solvent delay. In full scan mode, EI mass spectra were obtained at 70 eV ionization voltages covering m/z 40–650. The temperatures of the transfer line and ion source were adjusted to 260 and 250 °C, respectively. Identification of phytochemical compounds was carried out using the Xcalibur data system (version 3.0) of GC-MS^41,42^.

### Methylation of lipids in weeping willow leaves and the analysis by GC-FID

Weighed out into a conical flask with 10 mL of concentrated HCl, 10 g of milled weeping willow leaves were heated in a water bath until the sample was completely dissolved. After adding 30 mL of diethyl ether to the mixture and giving it a good shake, the fats were extracted. The extract was then collected into a weighted flask, and the layers were allowed to separate. After three further extractions, the solvent was removed by distillation, and the fat was dried at 100 °C, cooled, and weighed^43^.

For the methylation of the extracted lipids from the leaves, a sample of 50 mg of lipids was weighed in a tube. Three chemicals were then added to each tube: 50 mL of the mixture of 1 mL of concentrated sulfuric acid, 100 mL of methanol, and 2 mL of benzene. The tube was sealed completely and placed in a water bath at 90 °C for an hour and a half. After cooling the tube, 8 mL of water and 5 mL of petroleum ether were added. The ethereal layer was then separated and evaporated after the tube was vigorously agitated^44^. Following their preparation, the fatty acid methyl esters were examined using gas-liquid chromatography (GC) analysis (HP; Hewlett Packard, 6890 GC) with flame-ionization detection (GC-FID) according to matching their retention times with standard fatty acids (C_2_-C_25_) chromatographed under the same conditions^45,46^. This was done using the parameters and conditions shown in Table 1^47,48^.

**Table 1:** Condition for analysis of methylated fatty acids by gas chromatography with flame-ionization detection (GC-FID).

Device model	HP (Hewlett Packard) 6890 GC		
Column	HP-5 (5% diphenyl, 95% dimethyl polysiloxane), 30 m, 0.32 mm. ID, 0.25 μm film thickness.		
Carrier gas/gas flow rate	Nitrogen (1 mL/min).		
Detector/temperature	FID (Flame Ionization Detector)/250°C.		
Injector temperature, Injection volume	220 °C, 2 µL in a splitless mode.		
Oven program	Initial Temp. 150 °C for 2 min.		

Ramps	Rate °C/min	Final Temp. °C	Hold time
1	10	200	-
2	5	250	9 min.

### Insecticidal **activity of the tested materials**

A stock solution of each HE was prepared in distilled water (DW) with Triton X-100 (0.05%) and dimethyl sulfoxide (0.1% DMSO) as an emulsifier. A basic stock solution of each HE (10000 mg/L) was prepared by adding 1 mL of the HE to a volumetric flask and completing it to 100 mL using DW containing Triton X-100 (0.05%) and dimethyl sulfoxide (0.1% DMSO) as an emulsifier and then shacked carefully several times. Five concentrations of 25, 50, 100, 500, and 1000 mg/L were prepared from stock solutions of HEs by adding 0.25, 0.5, 1, 5, and 10 mL of stock solution in a volumetric flask and completing 100 mL of DW.

The insecticidal activities of the three plant HEs and the insecticide mospilan on *T. ricini* and *B. tabaci* were checked under greenhouse conditions of 27 ± 2 °C, and 65 ± 5% RH. Five concentrations of 25, 50, 100, 500, and 1000 mg/L from each HE were prepared and the insecticidal effect was checked by spraying the uninfested seedlings of tomato and castor (5–6 leaves) until runoff. Two groups were used as controls, the first one plants sprayed with DW and another group sprayed with combined TritonX-100 (0.05%) and DMSO (0.1%). Treatments and controls were replicated three times. The treated seedlings were allowed to dry for 2 h in the shadow, and then 20 adult whiteflies per replicate were exposed using the aspirator tube to the treated and control seedlings covered with glass cages with muslin in the upper opening. The adult mortality was determined after 48 h of treatment. On the lower surface of the tomato or castor leaves, alive whitefly adults were counted. On the other hand, the dead insects can be seen over the potting soil where they fall. The adult mortality was calculated using the Biostat version 2.1 computer program for Probit analysis. Figure 1 shows the insecticidal activity of the HEs and the insecticide mospilan on *T. ricini* and *B. tabaci*.

### Joint toxic effect of the tested plant extracts in combination with mospilan against *Bemisia tabaci* and *Trialeurodes ricini* adults

The effects of the tested HEs in combination with the conventional insecticide mospilan were estimated. Both insecticide and plant extracts were mixed and applied as the LC_25_ values of mospilan were mixed with each of the LC_25_ values of the tested HEs. The adult mortality was determined as previously mentioned for the bioassay test. Co-toxicity factors (CTFs) were calculated as follows^47^:

$$\:\text{C}\text{T}\text{F}=\frac{\text{O}\text{b}\text{s}\text{e}\text{r}\text{v}\text{e}\text{d}\:\text{\%}\:\text{m}\text{o}\text{r}\text{t}\text{a}\text{l}\text{i}\text{t}\text{y}-\text{e}\text{x}\text{p}\text{e}\text{c}\text{t}\text{e}\text{d}\:\text{\%}\:\text{m}\text{o}\text{r}\text{t}\text{a}\text{l}\text{i}\text{t}\text{y}}{\text{E}\text{x}\text{p}\text{e}\text{c}\text{t}\text{e}\text{d}\:\text{\%}\:\text{m}\text{o}\text{r}\text{t}\text{a}\text{l}\text{i}\text{t}\text{y}}\times\:100$$

The expected mortality was calculated for each mixture of two materials by adding the mortalities of each material used in the mixture. The mixtures were categorized according to the CTFs as follows: CTFs < -20 meant antagonism; CTFs between − 20 and + 20 meant additive effect; and CTFs ≥ + 20 meant potentiation^49^.

### Biochemical assays

#### Homogenate preparation, protein content, and in vivo inhibition of AChE

Whitefly adults were collected after 48 h of treatment by LC_50_ values of mospilan alone and its mixtures with the tested extracts. Samples were homogenized with 10 volumes (w/v) of ice-cold 0.1 M phosphate buffer (pH 8.0) using a polytron homogenizer (Tekmar tissumizer) for 60 s. The homogenate was centrifuged for 30 min at 4 °C using a Janetzki K23 cooling centrifuge at 5000 rpm. The obtained supernatants were used for measuring protein content and the activities of AChE. The protein content was determined by using Bovine Serum Albumin^50^.

The activity of AChE was determined according to the colorimetric method^51^. One and a half grams of whole insects were homogenized in 7 mL of phosphate buffer (0.1 M, pH 8). The supernatant containing AChE was filtered through glass wool. Treated and control assays were corrected by blanks for non-enzymatic hydrolysis. Each assay was done in triplicate. The level of AChE activity was estimated by a PharmaSpec UV-1700 Shimadzu Spectrophotometer set at 412 nm after 10 min. The percentage of in vivo inhibition was calculated concerning the activity in the absence of treatments using the following formula:

$${\text{AChE inhibition percentage }}\left( {{\text{I }}\% } \right) = \left[ {{\text{1}} - {\text{SAT}}/{\text{SAC}}} \right] \times {\text{1}}00$$

#### Repellent effects of the tested materials on *Bemisia tabaci* adults (choice test)

Under greenhouse conditions, the repellency effect of the tested HEs was investigated using the choice test design. Spraying uninfested tomato seedlings with serial concentrations of HEs 0.05, 0.1, 0.5, 1, and 2% until runoff was done and compared with the two controls and replicated three times. The treated seedlings were allowed to dry for 2 h in the shadow, and then the treated and control plants were put together into insect cages. The arrangement of plants was completely randomized. Approximately 2500 adults were released into the greenhouse, and left for 48 h. After this time, plants were carefully extracted from the cages, and the number of adults/plants was carefully counted in the early morning as the insect’s movement is very little and slow, and we carefully counted them in small tubes (100 per tube). The adult repellency percentages were calculated as follows^52^:

$${\text{The repellency index (RI) = [(C}} - {\text{T) / (C}} + {\text{T)] }} \times {\text{100}}$$

Where, C = adults number in control, T = the number of adults in the treatment.

### Statistical analysis

Statistical analysis of the obtained data and all the probable comparison combinations were analyzed in a randomized complete blocked design (RCBD) by using analysis of variance (ANOVA) with the SAS procedure (SAS. User Guide: Statistics (Release 8.02); SAS Institute: Cary, NC, USA, 2001). The comparison among means was done using the least significant difference (LSD) at the 0.05 level of probability. The adult mortality was calculated using the Biostat version 2.1 computer program for Probit analysis.

### Ethics approval and consent to participate

This study is complied with relevant institutional, national, and international guidelines and legislation. This study does not contain any studies with human participants or animals performed by any of the authors.

## Results and discussion

### **Chemical** composition **of thyme**,** garlic**,** and weeping willow extracts**

Table 2 provides an overview of the primary chemicals identified for each HE from thyme, garlic, and weeping willow, while other compounds were detected and characterized chemically.

**Table 2:** Chemical composition of the *n*-hexane extracts of *Thymus vulgaris* leaves, *Allium sativum* bulbs, and *Salix babylonica* leaves by using gas chromatography-mass spectrometry apparatus.

Main compounds	Match factor	% Area by GC
Thyme (*Thymus vulgaris* L.)		
Thymol	901	9.53
Geranyl-α-terpinene	789	5.92
Oleic acid	847	5.18
Thymol methyl ether	956	4.96
Palmitic acid methyl ester	926	3.25
Palmitic acid	934	3.05
Tetradecane	923	2.96
Methyl eugenol	933	2.84
6-Methyltetralin	750	2.44
Z-α-trans-Bergamotol	821	2.44
2-Methyl-trans-decalin	858	2.43
1-Methyl-3-(2-methylpropyl)-cyclopentane	879	2.16
Total identified	-	99.89
Garlic (*Allium sativum* L.)		
Diallyl sulfide	774	6.39
Allyl tetrasulfide	909	5.71
Trisulfide, di-2-propenyl (Allyl trisulfide)	920	4.12
Dibenzofuran	902	3.41
Dibenzothiophene	729	3.05
Diallyl disulphide	742	2.71
2-Aminoethanethiolsulfuric acid	777	2.51
1-Octadecanesulphonyl chloride	787	2.45
2-Vinyl-4 H-1,3-dithiine	845	2.38
2-Butyl-1-octanol	848	2.25
2-Methylnaphthalene	933	2.18
Farnesane	895	2.00
Isonaphthofuran	943	2.00
Total identified	-	98.2
Weeping willow (*Salix babylonica* L.)		
6-Phenyltridecane	921	10.28
6-phenyldodecane	918	9.00
5-Phenyldodecane	896	8.69
2-Phenyldodecane	931	6.80
5-Phenyltridecane	913	6.64
4-Phenyldodecane	909	6.37
2-phenylundecane	885	5.46
5-phenylundecane	922	5.35
3-phenyldodecane	880	5.30
2-Phenyltridecane	930	5.00
4-Phenyltridecane	865	4.77
Heneicosane	878	4.02
Total identified	-	99.97

The main components found in the *n*-hexane extract (HE) of thyme (*T. vulgaris*) leaves were thymol (9.53%), geranyl-α-terpinene (5.92%), oleic acid (5.18%), thymol methyl ether (4.96%), palmitic acid (3.05%), and palmitic acid methyl ester (3.25%). Prior research revealed that the volatile oil obtained through steam distillation (sample dilution: 1% in *n*-hexane) contained high concentrations of *p*-cymene (30.53%) and thymol (30.86%), a monoterpene phenol derivative; by hexane extraction (sample dilution: 1% in n-hexane), the corresponding percentages were 1.01% and 0.81%, respectively^53^. The HE of thyme leaves contained thymol (40.86%), o-thymol (46.66%), thymol acetate (0.42%), and additional chemicals such as linalyl anthranilate (1.06%), *n*-hexadecanoic acid (0.64%), and α-linolenic acid (0.64%) and *α*-terpineol (0.76%)^54^. Thymol has been recognized as an active agent with several bioactivities, including insect repellents^55,56^.

The main identified compounds in the HE from garlic bulbs (*A. sativum*) were diallyl sulfide (6.39%), allyl tetrasulfide (5.71%), trisulfide, di-2-propenyl (allyl trisulfide) (4.12%), dibenzofuran (3.41%), dibenzothiophene (3.05%), diallyl disulphide (2.71%), and 2-aminoethanethiolsulfuric acid (2.51%). Insect toxicity was seen in the vapor phase due to the active ingredients in garlic HE, including dimethyl disulphide, diethyl trisulphide, di-n-propyl disulphide, allyl disulphide, diallyl tri-sulphide, diallyl disulphide (6.03%), 9-methyl-nonadecane, nonadecane, 2-methyl-tetradecane, octadecane, 2-hexyl-1-decanol, dodecanoic acid, hex-3-enyl ester (2.75%), and allyl thiosulphinates^57–59^. The main compounds in the HE from weeping willow (*S. babylonica*) leaves were 6-phenyltridecane (10.28%), 6-phenyldodecane (9.00%), 5-phenyldodecane (8.69%), 2-phenyldodecane (6.80%), 2-phenyltridecane (5.00%), 5-phenyltridecane (6.64%), and 4-phenyldodecane (6.37%). The majority of these chemicals were also found in the fruit HEs of *Ziziphus spina-christi* (L.) Desf. and *Phytolacca dioica* L^41^. The volatile extract from *S. babylonica* deterred the female beetles of *Monochamus alternatus* Hope (Coleoptera: Cerambycidae) from oviposition to different degrees^60^.

As shown in Table 3, the chemical composition of methylated fatty acids in the HE from weeping willow leaves was stearic (49.77%) and palmitic (14.96%) followed by arachidic (11.14%), 14-pentadecanoic (10.6%), and margaric (10.51%). Tritetracontane, octadecenoic acid-1,2,3-propanetriyl ester, hexadecanoic acid-methyl ester, and 1,3-dioxane-4-(hexadecyloxy)-2-pentadecyl, 9-octadecenoic acid, and aliphatic hydrocarbons such as nonadecane and hexatriacontane were identified in *S. babylonica* leaf extract^61^. The essential oils from the *S. babylonica* samples collected in Egypt included *α*-pinene, *β*-cedrene, salicylaldehyde, *cis*-4-hexen-1-ol, linalool, and 1,2-cyclohexanedione as the main components. On the other hand, the main chemical components of the *S. babylonica* samples collected in Vietnam were camphene, 2-(4-methyl-3-cyclohexen-1-yl)-2-propanol, 3,7-dimethyl-1,6-octadien-3-ol, geranyl acetate, α-humulene, pentacosane, trans-carvone oxide, thymol, trans-caryophyllene, *α*-cadinol, and farnesol^62^.

**Table 3:** Phytochemicals of methylated fatty acids presented in the *n*-hexane extract from *Salix babylonica* leaves by gas chromatography with flame-ionization detection.

Compound	Percentage of fatty acid (%)	Retention time (min)
Myristic acid (Tetradecanoic acid)	2.94	7.23
14-Pentadecanoic acid	10.6	7.99
Palmitic acid (Hexadecanoic acid)	14.96	9.23
Margaric acid (Heptadecanoic acid)	10.51	10.58
Stearic acid (Octadecanoic acid)	49.77	11.99
Arachidic acid (Eicosanoic acid)	11.14	15.08

### Insecticidal **activity of the tested extracts and mospilan against whiteflies adult**

The insecticidal activity of garlic, thyme, and weeping willow HEs against *T. ricini* and *B. tabaci* adults was evaluated. Nonetheless, the toxicity of mospilan (a commercial insecticide) was evaluated for a comparative toxicological study. The obtained data illustrated in Tables 4 and 5 proved that the toxicity of all tested HEs was more potent against *B. tabaci* (as a susceptible strain) than *T. ricini* (a field strain).

**Table 4:** Comparative toxicity of tested materials against *Bemisia tabaci* adults after 48 h from treatment.

Treatments	LC_25_ (mg/L)	LC_50_ (mg/L)	Lower limit	Upper limit	Slope ± SE*
Garlic HE	5.21	52.73	23.39	89.13	0.671 ± 0.13
Thyme HE	14.32	108.10	65.44	169.27	0.768 ± 0.13
Weeping willow HE	3.68	54.61	20.18	99.30	0.576 ± 0.12
Mospilan	2.57	6.68	5.17	8.57	1.623 ± 0.23

*SE = standard error; HE: *n*-hexane extract.

LC_25_ = Lethal concentration 25 = the concentration of the chemical that kills 25% of tested insects during the observation period.

LC_50_ = Median lethal concentration = the concentration of the chemical that kills 50% of tested insects during the observation period.

**Table 5:** In vivo inhibition of acetylcholinesterase (AChE) activity of *Bemisia tabaci* and *Trialeurodes ricini* adults as affected by LC_50_ values of mospilan alone and its mixtures with the tested extracts.

Treatment	AChE activity (µmol/min/mg protein)		I %	
	*B. tabaci*	*T. ricini*	*B. tabaci*	*T. ricini*
Control	32.20^a^	36.23^a^	-	-
Garlic HE + Mospilan	16.70^c^	23.42^b^	48.14	35.36
Thyme HE + Mospilan	13.53^d^	20.24^c^	57.97	44.13
Weeping willow HE + Mospilan	12.92^d^	16.83^d^	59.88	53.54
Mospilan	18.43^b^	24.76^b^	42.75	31.66

Means within the same column followed by the same letters are not significantly different at *P* ≤ 0.05. Three replicates per treatment were used. AChE inhibition percentage (I %) = [1 - SAT/SAC] × 100, where SAT is the specific activity of the enzyme in the treatment and SAC is the specific activity of the enzyme in the control.

Mospilan recorded the highest toxicity against both *T. ricini* and *B. tabaci* adults (LC_50_ values were 6.13, and 6.68 mg/L, respectively), followed by garlic and weeping willow HEs against *B. tabaci* then thyme and garlic HEs against *T. ricini* (LC_50_ values were 52.73, 54.61, 91.13, and 92.13 mg/L, respectively). On the other hand, thyme HE showed less toxicity against *B. tabaci*, and weeping willow HE had less toxicity against *T. ricini* with LC_50_ values of 108.10 mg/L (Table 4) and 163.99 mg/L (Table 6), respectively. The differences in an insect’s susceptibility to the tested materials may be due to the differences between the species as well as the host and insect resistance.

**Table 6:** Comparative toxicity of tested materials against *Trialeurodes Ricini* adults after 48 h from treatment.

Treatments	LC_25_ (mg/L)	LC_50_ (mg/L)	Lower limit	Upper limit	Slope ± SE*
Garlic HE	19.73	92.13	61.45	131.31	1.008 ± 0.136
Thyme HE	12.40	91.13	53.92	157.97	0.799 ± 0.176
Weeping willow HE	52.92	163.99	125.18	217.35	1.373 ± 0.144
Mospilan	1.49	6.13	4.12	8.75	1.099 ± 0.214

*SE = standard error; HE: *n*-hexane extract.

LC_25_ = Lethal concentration 25 = the concentration of the chemical that kills 25% of tested insects during the observation period.

LC_50_ = Median lethal concentration = the concentration of the chemical that kills 50% of tested insects during the observation period.

The toxic properties of the tested extract were previously reported by Aslan et al.^63^, who mentioned that, some of the tested extracts, including thyme (*T. vulgaris*), showed high toxicity against *B. tabaci* adults. According to Yang et al.^64^, as compared to controls, the cumulative effect of thyme reduces the survival rate of *B. tabaci* by 73.4%, 79.0%, and 58.2% following treatment of eggs, nymphs, and pupae, respectively. The cumulative survival rates of female *B. tabaci* treated with *T. vulgaris* were 46.4% lower in no-choice testing. Thyme and garlic extracts caused significant suppression of the *B. tabaci* population, with 100% mortality at a rate of 2.4 mL/cm^3^^65^. Thyme extract showed effective toxicity against eggs, 3rd instar nymphs, and adults of *B. tabaci*, reducing the percentage of *B. tabaci* populations by about 81.9%^66^. The insecticidal activities of the essential oils from thyme and peppermint for controlling the greenhouse whitefly, *Trialeurodes vaporariorum* Westwood (Hemiptera: Aleyrodidae), proved that thyme was more effective than peppermint (*Mentha × piperita* L.)^67^.

The current insecticidal activity results of garlic HE were consistent with previous findings^58,68^. The garlic extract showed insecticidal activity on stored product pests^68^. Further, the extract observed insecticidal activity on the cotton leafworm, *Spodoptera littoralis* (Boisd.) (Lepidoptera: Noctuidae)^57^. Trisulfide, di-2-propenyl, and diallyl disulfide are the main compounds in garlic bulb extract with significant insecticidal activity against the stored cowpea seeds, *Callosobruchus maculatus* (Fab.) (Coleoptera: Chrysomelidae)^69,70^. It was observed that dimethyl and diallyl thiosulfinates were more toxic than disulfur against *C. maculates*, *Sitophilus oryzae* L. (Coleoptera: Curculionidae), *S. granaries* L. (Coleoptera: Curculionidae), *Ephestia kuehniella* Zeller (Lepidoptera: Pyralidae), and *Plodia interpunctella* (Hübner) (Lepidoptera: Pyralidae)^71^.

Furthermore, garlic extract, which has two main compounds diallyl tri-and disulfide, exhibited strong fumigant toxicity against *B. tabaci*^72^. The efficacy of the essential oils of *Piper marginatum* Jacq. and *Mansoa alliacea* (Lam.) A.H.Gentry against *B. tabaci* nymphs was demonstrated by their reported LC_50_ values of 9.39 µL/mL and 10.99 µL/mL (corresponding to 9390–10,990 mg/L)^73^.

### Joint toxic action of mospilan with plant extracts against *B. Tabaci* and *T. Ricini* adults

The effects of applying mixtures of the LC_25_ of mospilan with the LC_25_ of each tested plant’s HEs against *T. ricini* and *B. tabaci* adults were determined. The results showed that all mixtures of mospilan with each of the tested three plant HEs resulted in a potentiating toxic effect with Co-Toxicity Factors (CTFs) ranging between 21.47 for garlic HE + mospilan against *B. tabaci* and 37.65 for weeping willow HE + mospilan against *T. ricini* (Table 7). Generally, the joint toxic effect of mospilan with the tested plant HEs against *T. ricini* was higher than the effect against *B. tabaci.*

**Table 7:** Combined toxicity of mospilan (LC_25_) with tested plant extracts (LC_25_) against the whiteflies *Bemisia tabaci* and *Trialeurodes ricini* adults.

Mixture	Tested insect	Observed mortality %	Expected mortality %	Co-toxicity factor (CTF)
Garlic HE + Mospilan	*B. tabaci*	71.67	59.00	21.47
	*T. ricini*	70.00	57.33	22.10
Thyme HE + Mospilan	*B. tabaci*	75.00	59.67	25.69
	*T. ricini*	80.00	58.66	36.38
Weeping willow HE + Mospilan	*B. tabaci*	78.33	61.33	27.72
	*T. ricini*	81.67	59.33	37.65

The highest potentiating toxic effect was obtained after 48 h of exposure of whitefly adults to mospilan mixed with weeping willow HE; the CTFs were 27.72 and 37.65 against *B. tabaci* and *T. ricini*, respectively. On the contrary, garlic HE, when mixed with mospilan, recorded the lowest potentiating toxic effect, where the CTFs were 21.47 and 22.10 against *B. tabaci* and *T. ricini* adults, respectively (Table 7). It is possible to state that the potentiating toxic effect is due to the broad-spectrum toxic properties of the tested extracts. Combined insecticides with plant extracts could increase the efficiency of toxicological action by achieving synergism, acting on various targets simultaneously, and decreasing the doses of insecticide alone. The objective of these findings is to mitigate the effects of conventional insecticides, like mospilan, on the environment and non-target organisms in fields by lowering their doses when combined with plant extracts.

All mixtures of Cetam 20%SL^®^ (Neonicotinoid Insecticide) with tested plant oils, cumin, thyme, and garlic resulted in an additive or potentiating effect after 48 h of *B. tabaci* exposure, and a higher potentiating effect was obtained with the mixture of cetam and thyme oil^39^. The binary mixtures of each of sulfoxaflor, flonicamid, and flometoquin compounds with lemongrass oil exhibited synergism in all combinations against *B. tabaci*, which observed mortalities ranging from 15.09 to 22.94% higher than expected for an additive effect^74^.

### Biochemical assays (*in vivo* inhibition of AChE activity)

*In vivo* inhibition of AChE activity of *B. tabaci* and *T. ricini* adults as affected by the LC_50_ values of mospilan alone and its mixtures with the tested plant HEs illustrated in Table 5. These outcomes are consistent with the tested HE’s synergistic action when combined with mospilan, as the combination with plant extracts significantly increased the inhibitory effect of the insecticide. Generally, *B. tabaci* was more sensitive to different treatments (as a susceptible strain) than *T. ricini* (a field strain). The highest AChE inhibitory effect (I%) was obtained after 48 h of exposure of whitefly adults to the mixture (mospilan + weeping willow HE); the I% was 59.88% and 53.54% against *B. tabaci* and *T. ricini*, respectively. On the contrary, no significant difference is recorded when mospilan mixed with garlic HE.

Several studies reported the neurotoxic effects of the extract by AChE inhibition or by blocking the octopamine receptors^17^. Thymol from *T. vulgaris* showed moderate inhibitory potential on AChE^75^. Another study observed that carvacrol was a more effective inhibitor than thymol on the AChE enzyme of *Drosophila melanogaster* Meigen^76^. For anesthetic in silver catfish, thymol exposure at 50 mg/L is preferable to carvacrol because it did not result in any mortality or interfere with AChE activity^77^. Previous investigations have also demonstrated that the sulfur compounds in extracts of *Allium* spp. may prevent insects’ acetylcholinesterase enzyme from activating^78,79^.

### Repellence or attractant effect of the tested plant extracts on ***B. tabaci*** adults

**Fig. 2:**
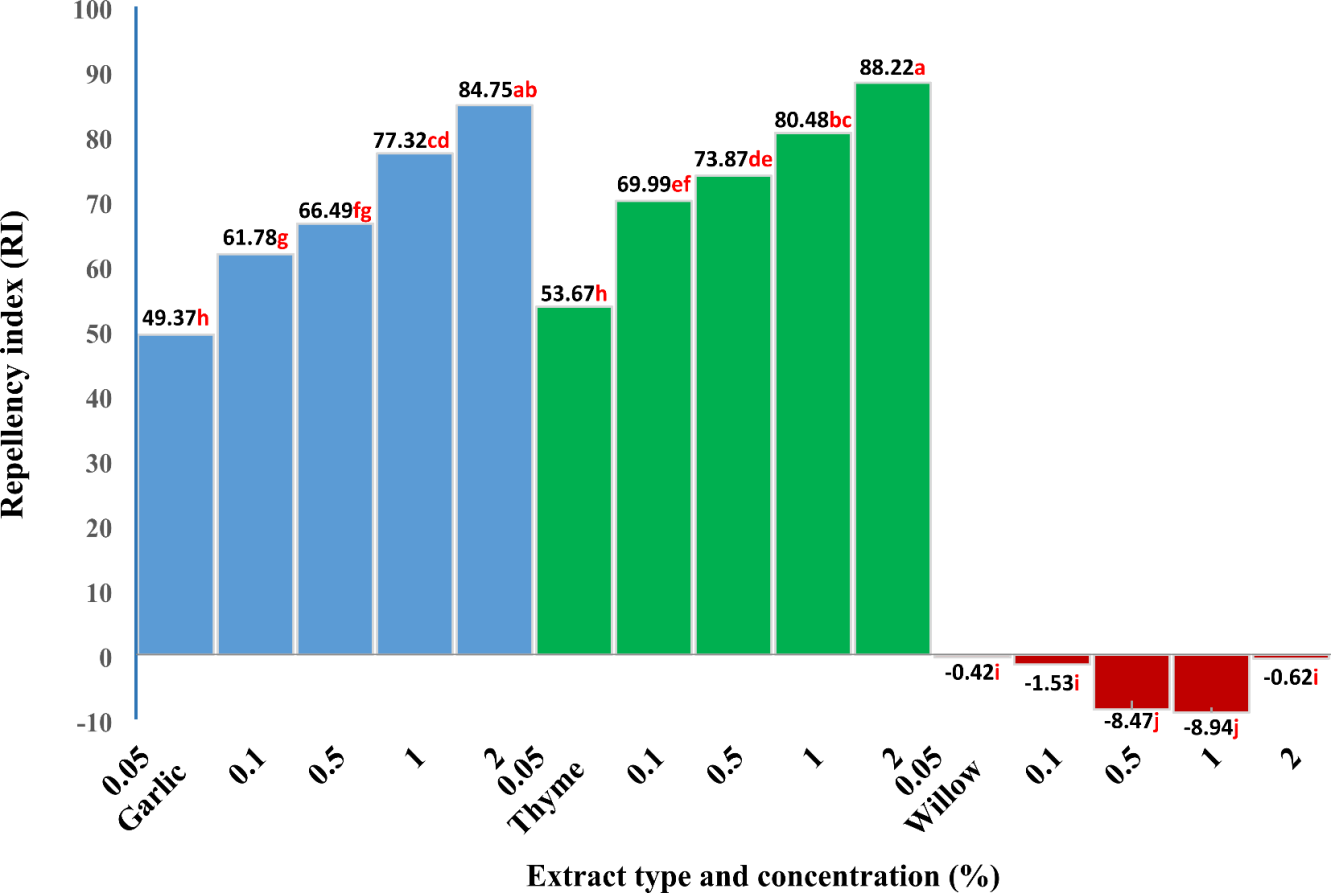
Repulsive effect of garlic, thyme, and willow extracts on *Bemisia tabaci* adults. Means with the letter(s) are not significantly different according to the least significant difference (LSD) at the 0.05 level of probability.

The repellent and attractive effects of the tested HEs on *B. tabaci* adults were investigated. The repellent and attractive effects of garlic, thyme, and weeping willow HEs were illustrated in Fig. 2 as a repellency index (RI). These results show significant repellency effects of garlic and thyme HEs on *B. tabaci* adults positively correlated with the extract’s concentration. Conversely, weeping willow HE had an attractant effect on *B. tabaci* adults, which was also correlated positively with the increase in the extract’s concentration, except at the highest concentration of 2%.

Generally, thyme HE showed the highest repellency effect at all tested concentrations compared to the corresponding garlic HE concentrations. The study by Emilie et al.^80^ showed that among 20 plant extracts, the seven most toxic and irritant products against *B. tabaci* were aframomum, citronella, litsea, geranium, dill, cinnamon, and savory; however, thyme extract recorded repellent and irritant effects on the behavior of *B. tabaci*. In choice tests, 59.0% fewer eggs were deposited by *B. tabaci* on average on *T. vulgaris* oil-treated plants than on controls^64^. Thyme and garlic oils effectively prevented *B. tabaci* adults either from feeding or laying eggs^39^. Garlic extract has insecticidal qualities and exhibits significant toxicity and repellency to a variety of pest species, according to Bardin et al.^81^. Garlic extract is harmless for the environment, bio-enemies, and consumers of cotton goods, although it is not as efficient or quick to act as synthetic pesticides^82^. To obtain the repellent effect of garlic, as a volatile plant, against the abovementioned insects, El-Shamy and Abd El-Aty^83^ intercropped Balady garlic or Sids-40 garlic with faba bean. The *B. tabaci* population density was reduced by 59.92% and 35.26%, respectively.

In comparison with the control (100%) and the weeping willow extract (0.01% concentration), the maximum insect attraction value of 28.79% indicated that the highest percentage of insects were attracted to the weeping willow extract^84^. Guided by a repellency index (RI) value, the highest repellent effect was recorded by thyme and garlic HEs at the highest concentration of 2%, with no significant difference between them, with RI 88.22% and 84.75%, respectively. Alternatively, weeping willow HE achieved the highest attractant effect at concentrations of 1% and 0.5% with RIs − 8.94 and − 8.47%, respectively. Differences in the attraction or repellency effects of the tested plant extracts could result from differences in their chemical composition. Furthermore, the insecticidal and repellent efficacy of selected botanical oils, including garlic and thyme, against the whitefly *B. tabaci* was performed^85^. Additionally, and with the same manner, a three botanical oils (patchouli, thyme, and neem) could be effectively developed and incorporated into IPM packages for the management of whiteflies^85^.

The limitation of this study can be drawn in the choice test under greenhouse conditions, which needs a large number of castor seedlings and a huge number of *T. ricini* adults, which was difficult to provide and implement. This needs more research and studies in the future.

It is important to keep in mind that bioactivity data obtained under greenhouse conditions may not always be equal to in vivo toxicity. Thus, this study opens the door for further research on the effects of plant extracts over an extended period, or shelf life when mixed with an insecticide.

## Conclusion

The present study proved the insecticidal activity as well as potentiating toxic effects (in combination with mospilan) of selecting extracts from thyme leaves, garlic bulbs, and weeping willow leaves against two whitefly species and the repellent or attractant properties of the same extracts on *B. tabaci* adults. The present results confirm the possibility of using the tested plant extracts as effective and environmentally sustainable bio-insecticides for controlling whiteflies, either directly due to their insecticidal effect or indirectly through the repellent or attractant effect. This is in addition to their potentiating toxic effect when mixed with conventional insecticides, which enable reducing the doses of conventional insecticides such as mospilan and thus reducing the impact of these insecticides on the environment and nontarget organisms in fields.

## Data Availability

All data generated or analyzed during this study are included in this published article.
